# Development of optimization method for uniform dose distribution on superficial tumor in an accelerator-based boron neutron capture therapy system

**DOI:** 10.1093/jrr/rrad020

**Published:** 2023-04-26

**Authors:** Akinori Sasaki, Naonori Hu, Nishiki Matsubayashi, Takushi Takata, Yoshinori Sakurai, Minoru Suzuki, Hiroki Tanaka

**Affiliations:** Graduate School of Engineering, Kyoto University, Kyoto University Katsura Campus, Kyoto Nishikyo-ku, Kyoto 615-8246, Japan; Particle Radiation Oncology Research Center, Institute for Integrated Radiation and Nuclear Science, Kyoto University, 2-Asashiro-Nishi, Kumatori-cho, Sennan-gun, Osaka 590-0494, Japan; Kansai BNCT Medical Center, Educational Foundation of Osaka Medical and Pharmaceutical University, Daigakumachi, Takatsuki, Osaka 569-0801, Japan; Graduate School of Engineering, Kyoto University, Kyoto University Katsura Campus, Kyoto Nishikyo-ku, Kyoto 615-8246, Japan; Particle Radiation Oncology Research Center, Institute for Integrated Radiation and Nuclear Science, Kyoto University, 2-Asashiro-Nishi, Kumatori-cho, Sennan-gun, Osaka 590-0494, Japan; Particle Radiation Oncology Research Center, Institute for Integrated Radiation and Nuclear Science, Kyoto University, 2-Asashiro-Nishi, Kumatori-cho, Sennan-gun, Osaka 590-0494, Japan; Particle Radiation Oncology Research Center, Institute for Integrated Radiation and Nuclear Science, Kyoto University, 2-Asashiro-Nishi, Kumatori-cho, Sennan-gun, Osaka 590-0494, Japan; Particle Radiation Oncology Research Center, Institute for Integrated Radiation and Nuclear Science, Kyoto University, 2-Asashiro-Nishi, Kumatori-cho, Sennan-gun, Osaka 590-0494, Japan

**Keywords:** accelerator-based boron neutron capture therapy, uniform thermal neutron flux, intensity-modulated irradiation, optimization method, uniform dose distribution

## Abstract

To treat superficial tumors using accelerator-based boron neutron capture therapy (ABBNCT), a technique was investigated, based on which, a single-neutron modulator was placed inside a collimator and was irradiated with thermal neutrons. In large tumors, the dose was reduced at their edges. The objective was to generate a uniform and therapeutic intensity dose distribution. In this study, we developed a method for optimizing the shape of the intensity modulator and irradiation time ratio to generate a uniform dose distribution to treat superficial tumors of various shapes. A computational tool was developed, which performed Monte Carlo simulations using 424 different source combinations. We determined the shape of the intensity modulator with the highest minimum tumor dose. The homogeneity index (HI), which evaluates uniformity, was also derived. To evaluate the efficacy of this method, the dose distribution of a tumor with a diameter of 100 mm and thickness of 10 mm was evaluated. Furthermore, irradiation experiments were conducted using an ABBNCT system. The thermal neutron flux distribution outcomes that have considerable impacts on the tumor’s dose confirmed a good agreement between experiments and calculations. Moreover, the minimum tumor dose and HI improved by 20 and 36%, respectively, compared with the irradiation case wherein a single-neutron modulator was used. The proposed method improves the minimum tumor volume and uniformity. The results demonstrate the method’s efficacy in ABBNCT for the treatment of superficial tumors.

## INTRODUCTION

Boron neutron capture therapy (BNCT) is a type of radiation therapy that uses charged particles: alpha particles and ^7^Li nuclei emitted as nuclear by-products in reactions between ^10^B and thermal neutrons [1, 2]. These charged particles have high-linear energy transfer characteristics and a short range that corresponds to the approximate diameter of the cancer cells of interest. Therefore, when ^10^B accumulates in cancer cells, it selectively kills them.

In recent years, neutron sources for BNCT have transitioned from reactors to accelerators. Accelerator-based neutron sources can deliver epithermal neutrons with higher energies than reactor-based neutron sources for the treatment of deep-seated tumors.

A cyclotron-based epithermal neutron source has been developed for BNCT applications [3]. Based on the clinical trial results [4, 5], BNCT has been covered by insurance as a treatment method for unresectable, locally advanced or locally recurrent head and neck cancers in Japan since June 2020. Therefore, the number of facilities that perform BNCT is expected to increase in the future. In addition, BNCT applications are expected to expand for skin cancer, such as angiosarcoma [6] and malignant meningioma [7].

Epithermal neutrons are not suitable for the treatment of superficial tumors because the tumors need to be irradiated with thermal neutrons. In addition, it is difficult to uniformly distribute thermal neutrons onto tumors that spread over a broad area. Therefore, uniformly irradiating these tumors with a sufficient dose is difficult. To apply accelerator-based BNCT to the treatment of superficial tumors, it is necessary to develop a method that efficiently moderates epithermal neutrons and irradiates the tumors with uniform and intense thermal neutrons.

Previous studies have demonstrated that a bolus placed on a patient’s body can be effectively used to uniformly irradiate a region with an approximate diameter of 5 cm with thermal neutrons [8]. A single-neutron modulator, such as polyethylene (PE), can be placed inside a collimator to generate thermal neutrons [9]. However, these methods do not provide sufficient tumor doses for relatively large tumors owing to the reduced thermal neutron flux at the tumor edges. Unlike X-rays and proton beams, the epithermal neutron beams used in BNCT are not parallel; therefore, they are strong in the middle and become less intense toward the edges. In addition, it is difficult to form a flat thermal neutron distribution in the irradiated area simply by installing a modulator or bolus of a certain thickness to moderate energy to thermal neutron regions. To address this limitation, an intensity-modulated irradiation method that generates a uniform dose distribution by overlapping the irradiation field with a circular intensity modulator placed inside a collimator has been reported [10]. This approach was shown to be effective for symmetrical tumors with approximate diameters of 100 mm that spread over a wider area. For clinical applications, it is necessary to extend the intensity-modulated irradiation method to various non-symmetrical tumor shapes. In previous methods, the dose distribution was evaluated by manually changing the shape of the intensity modulator, which was a time-consuming process. Therefore, in this study, we developed a method that can automatically determine the optimal shape of an intensity modulator to generate a uniform tumor dose distribution for various tumor shapes. This method was developed, in part, by using the simulation environment for radiotherapy applications (SERA) [11], a treatment-planning software.

To evaluate the efficacy of this method, an intensity modulator with an optimized geometry was designed, and irradiation experiments were conducted using an accelerator BNCT system. This approach facilitated the automatic determination of a combination of intensity modulators and irradiation time ratios (irradiation time of each irradiation field divided by the total irradiation time) and can be applied in the clinical applications.

## MATERIALS AND METHODS

### Determination of intensity modulator shape and irradiation time ratio

SERA was used to perform iterative Monte Carlo neutron transport calculations to determine the optimal intensity modulator shape for a given tumor because of its short computation time. An accelerator-based neutron source was used as the neutron source. To create a uniform thermal neutron flux distribution for large tumors, increasing the collimator diameter as much as possible is effective. Because this study was primarily aimed at treating superficial tumors with the use of an accelerator-based neutron source, we used a 15-cm diameter collimator, the largest collimator diameter currently used in clinical practice. To minimize the number of intensity modulator changes, the maximum number of irradiation field combinations was set to two. The shape of the first intensity modulator was a PE disk (thickness = 2 cm, diameter = 15 cm) to enhance the thermal neutron flux on the skin’s surface for the entire irradiation field [9]. This irradiation field with a PE disk is called as irradiation field A (IF-A).

**Fig. 1:**
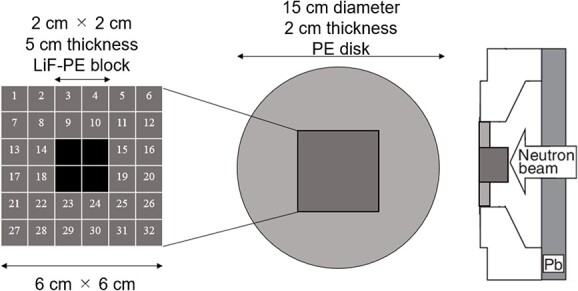
Schematic of intensity-modulator shape determination of IF-B.

The second irradiation field is called as irradiation field B (IF-B). The shape of the intensity modulator used in IF-B was determined as shown in Fig. 1.

First, the intensity modulator was made of the same material as the collimator, which could shield the epithermal neutrons of the treatment beam. The collimator material was PE, which was loaded with lithium fluoride (LiF-PE). LiF-PE contained ^6^Li, with a natural abundance ratio of 7.5%. The density of LiF-PE was 1.44 g/cm^3^. The thermal neutron flux at the edge of the irradiation field should be designed to be higher than that at the center. By overlapping IF-A and IF-B at an appropriate time ratio, the thermal neutron flux can be uniformly distributed in the irradiation field, and the dose distribution is expected to be improved. The intensity-modulated irradiation method used in this study required the modification of the intensity modulator in the collimator, but not the direction of irradiation or the patient’s body position. Therefore, for any tumor, the irradiation direction is set such that the center of the irradiation is at the center of the tumor. Therefore, in the intensity modulator used in IF-B, the center of the irradiation field was shielded by an LiF-PE block for any tumor shape. This resulted in a higher thermal neutron flux at the edges of the irradiation field than that at the center. IF-B had a 15 cm diameter and included a 2-cm thick PE disk with a 6- × 6-cm center that enabled the setting of an arbitrarily shaped LiF-PE block. To ensure accuracy when creating the blocks and to account for computation time when repeating Monte Carlo calculations, the minimum size of the blocks was set to a 1 cm mesh. As shown in Fig. 1, the smallest shielding pattern in the center was 2 × 2 cm. Therefore, this central region consisted of a 5-cm thick LiF-PE block. Previous studies have shown that a 5-cm thick LiF-PE is effective for shielding [10]. However, calculations were also performed for other thicknesses (e.g. 2, 3 and 4 cm) because a thinner LiF-PE may result in a better distribution depending on the shape of the tumor. A 1- × 1-cm LiF-PE block or a 1- × 1-cm PE block (thickness = 2 cm) was filled at Sites 1–32, as shown in Fig. 1. The grid size of the intensity modulator can be reduced to finely control the thermal neutron flux distribution. However, in the current method for determining the intensity modulator shape, the grid size directly affects the total calculation time. In addition, if the grid size is considerably small, gaps and neutron penetration will occur when considering the actual creation of the intensity modulator shape for irradiation tests. Therefore, a grid size of 1 cm was chosen to ensure accuracy in terms of calculation time and actual fabrication.

Considering the intensity modulators that could be designed using this approach, 106 different patterns were selected to improve the dose distribution. Criterion 1 represents the case where four LiF-PE blocks are placed at the center. We first created a pattern with blocks placed around this (8, 9, 10, 11, 14, 15, 18, 19, 22, 23, 24 and 25). The case where one block is placed (e.g. at 9) was considered first. Subsequently, the number of blocks to be placed was sequentially increased as two (e.g. at 9 and 10), three blocks (e.g. at 9, 10 and 15) and so on. Because assuming all the possible placement methods would require an enormous amount of computation time, we created a block arrangement that follows the assumed tumor shape. In this case, 64 different patterns were created.

Next, we considered the case where LiF-PE blocks were placed at four central and (8, 9, 10, 11, 14, 15, 18, 19, 22, 23, 24 and 25) positions as Criterion 2. Similar to Criterion 1, we created a pattern with blocks placed around this (1, 2, 3, 4, 5, 6, 7, 12, 13, 16, 17, 20, 21, 26, 27, 28, 29, 30, 31 and 32). In this case, 41 different patterns were created. Finally, we considered the case where LiF-PE blocks were placed at all locations (1–32), creating 106 different patterns.

A total of 424 different intensity modulator shapes of intensity modulators were investigated, with each pattern having a thickness of 2, 3 and 4 cm of LiF-PE. Source data were created to include the geometry of each intensity modulator. The source data also included the radiation beam data for an accelerator-based neutron source. All Monte Carlo calculations with 424 different intensity modulators were repeatedly performed, and a tool was created to automatically compile a list of parameters, such as minimum tumor dose and irradiation time, when overlapped with IF-A at irradiation time ratios of 1:2, 1:3, 1:4 and 1:5 for each of the 424 patterns. This tool was created by us with the use of Python and shell scripts. Dose calculations were performed for each input tumor shape using each prepared source data. The dose-calculation results were generated for the number of the prepared source data. These results were recalculated assuming the irradiation time ratios of IF-A and IF-B as 1:2, 1:3, 1:4 and 1:5. Finally, four different results were obtained for the irradiation time ratios of 1:2, 1:3, 1:4 and 1:5 for each source data. From these results, the minimum tumor dose was listed. To simplify calculations, the irradiation time ratio was assumed to be constant. A finer irradiation time ratio can improve the minimum tumor dose. Based on the list, the highest minimum tumor dose was chosen as the shape of the intensity modulator for IF-B and the irradiation time ratio for the intensity-modulated irradiation. In this study, the conditions that maximized the minimum tumor dose were considered to be optimal.

In this study, the optimal intensity modulator shape and irradiation time ratio were determined by inputting the shape of the tumor and by determining the beam direction. A tumor model was established for the treatment of superficial tumors using the BNCT accelerator system. The cases were defined as shallow or widespread. The tumor had a diameter of 100 mm and a thickness of 10 mm and was centered on the vertex of the human head. The collimator diameter was 15 cm; the largest diameter is used in clinical practice. We evaluated it in conditions like those in actual clinical practice aiming to clinical applications. Furthermore, the intensity modulator shape was based on the shape of a block mesh. Based on the above, we consider it important to re-evaluate whether it is feasible to deal with large tumors, such as those reported in [10].

**Fig. 2:**
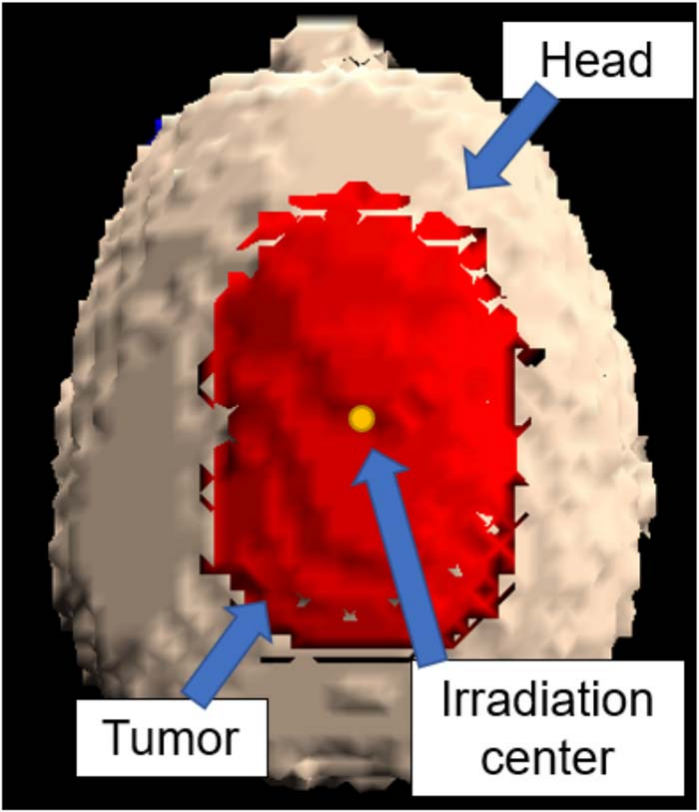
3D model of the head obtained using the SERA.

A 3D model of the head and tumor represented by SERA is shown in Fig. 2.

### Evaluation of tumor dose distribution

The irradiation time, relative biological effectiveness (RBE) equivalent dose to the tumor and the homogeneity index (HI) for intensity-modulated irradiation were compared with those for treatment using IF-A only. The advantages of this method, including the effectiveness of the combination of the intensity modulators, and the determination of the irradiation time ratio were evaluated. The irradiation conditions were as follows: normal skin dose, 12 Gy-eq [6]; brain dose, 15 Gy-eq; blood–boron concentration, 24 parts per million (ppm); normal skin and brain boron concentration, 24 ppm and T/B ratio, 3.5 for the tumor. The T/B ratio corresponds to the ratio of boron concentration in the tumor and blood–boron concentration. The RBE value of the hydrogen dose was assumed to be 2.4, the nitrogen dose was assumed to be 2.9 and the gamma dose was assumed to be 1.0 [4]. The compound biological effectiveness (CBE) values for the boron dose were set at 3.8 for tumors, 2.5 for normal skin and 1.34 for healthy brains. The dose to the tumor was calculated based on the aforementioned conditions, and the dose distribution was evaluated with the use of a dose-volume histogram. The prescribed dose of current BNCT is different from that in conventional radiotherapy. The absorbed dose in BNCT is the sum of the boron dose resulting from the reaction of boron with thermal neutrons, dose from gamma rays mixed in the neutron beam and the incidental non-boron dose resulting from the reaction of neutrons with the elements that constitute the body. To evaluate the dose to the tumor and surrounding normal tissue from these multiple absorbed doses, the equivalent dose is evaluated by multiplying each absorbed dose value by a factor (RBE or CBE) based on the biological effect ratio. The greatest difference in tumor dose and dose to normal tissue is in the boron dose. The thermal neutron flux and boron concentration in the tumor determine the boron dose. Knowing the distribution of boron concentration in the tumor is difficult during irradiation. Therefore, the boron concentration in the tumor is assumed by measuring the blood–boron concentration before and after the treatment and by using the ratio of the assumed blood–boron concentration to the boron concentration in the tumor (T/B ratio). However, currently, accurately estimating the tumor dose is difficult because the T/B ratio differs for each tumor tissue and patient. Therefore, the prescribed dose in current BNCT is defined by the tolerable dose of normal tissues. In this study, the prescribed dose was also determined by the maximum brain or skin dose. The HI was defined using the following equation:


$$ \mathrm{HI}=\frac{D_2-D}{D_{50}}, $$

where *D*_2_, *D*_50_ and *D*_98_ are the doses (Gy-eq) at which 2, 50 and 98% of the tumor volumes are irradiated, respectively. The ideal HI value is zero. The most ideal way to increase treatment efficacy is to make the difference between the lowest dose rate value in the tumor region and highest dose rate value in normal tissue as large as possible. However, as mentioned above, the boron concentration that determines the boron dose is only a predicted value. Furthermore, the distribution of boron concentration within the tumor is not actually uniform and may have a distribution. Therefore, we believe that the distribution of thermal neutrons should be homogenized to reduce the uncertainty factor in the tumor dose as much as possible. In addition, the lowest dose in the tumor region appears mainly at the tumor edge; this is due to the lower thermal neutron flux at the tumor margins. Therefore, increasing the thermal neutron flux at the edge is effective to increase the minimum dose. Thus, the uniformity of the thermal neutron distribution will increase.

A lower boron concentration in the blood results in a poorer tumor dose distribution. In addition, when the boron concentration in the skin increases, the irradiation time becomes shorter, and the minimum tumor dose is lowered. By evaluating these effects, we can show the superiority of this method compared with IF-A only. During the treatment, blood–boron concentrations can range from 12 to 37 ppm [4, 12]. Boron concentration changes affected the minimum tumor dose. Therefore, in this study, SERA was used to calculate the minimum tumor dose when the blood–boron concentration varied from 10 to 40 ppm. The effects of different blood–boron concentrations on the irradiation method were evaluated to examine the effectiveness of the intensity-modulated irradiation.

The skin/blood (S/B) ratio of normal skin can also be >1.0 [6]. When the S/B ratio of normal skin is higher, the maximum dose for normal skin defines the prescribed dose, which may shorten the irradiation time.Therefore, in this study, the minimum tumor dose was determined when the S/B ratio of normal skin varied from 1.0 to 1.5. The effects of changes in the S/B ratio of normal skin on the irradiation method were evaluated to examine the effectiveness of the intensity-modulated irradiation method.

### Irradiation test

We validated a method that can automatically derive the optimal shape of an intensity modulator. The intensity modulator was created, and irradiation tests were performed in an accelerator-based neutron source with the use of a head phantom. The head phantom was created using a 3D printer. The 3D model was based on published computed tomography images,[9, 13–16]. To measure the thermal neutron flux, a gold foil was placed on the phantom surface. The gold foil was placed at the center of the irradiation field, in the anterior–posterior (AP) and left temporal directions every 2.5 cm (total number of evaluation points = 10). The right side was omitted because of the symmetry of the head and neutron beam. This phantom was placed in front of the collimator and was irradiated by using IF-A and IF-B. In addition, the same irradiation experiment was performed using gold foil covered with Cd. The thermal neutron flux at the phantom surface was derived by measuring the induced activity of ^198^Au [8]. The uniformity index *u* is defined as a measure of the uniformity of the thermal neutron flux based on the following equations:


(1) \begin{equation*} u=\frac{\sum_1^7\left|100\times \left(1-\frac{\phi_{\mathrm{i}}}{\phi_{\mathrm{av}}}\right)\right|}{7}, \end{equation*}

(2) \begin{equation*} {\phi}_{\mathrm{av}}=\frac{\phi_1+{\phi}_2+{\phi}_3+{\phi}_4+{\phi}_5+{\phi}_6+{\phi}_7}{7}, \end{equation*}

where ${\phi}_{\mathrm{av}}$ is the average thermal neutron flux and ${\phi}_{\mathrm{i}}$ is the thermal neutron flux at each evaluation point.

**Fig. 3:**
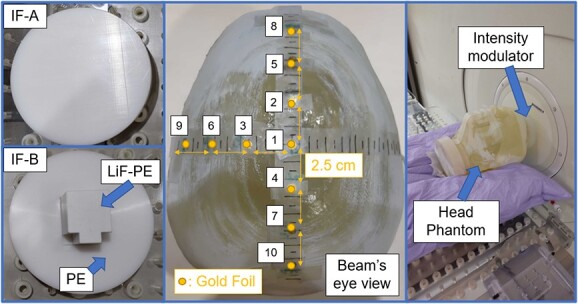
Intensity modulators and location of the gold foil in the setup during the irradiation test.

The thermal neutron flux at the evaluation points was calculated by using SERA to evaluate the validity of the developed method. The two types of intensity modulators, the location of the gold foil and the setup used during the irradiation tests, are shown in Fig. 3.

### Adaptation to other cases

The effectiveness of the developed method and intensity-modulated irradiation method was evaluated in two cases.

The first model was an angiosarcoma tumor in the right temporal cortex with a spatial range of 40 × 40 mm and a thickness of 20 mm. This case emulates a tumor shape similar to those typically treated with an accelerator neutron source [6]. The second model included tumors of asymmetrical shapes. The tumor size and thickness are the same as those in the Irradiation test subsection, but the tumor in this model had an asymmetrical shape.

## RESULTS

### Determination of intensity modulator shape and irradiation time ratio

**Fig. 4:**
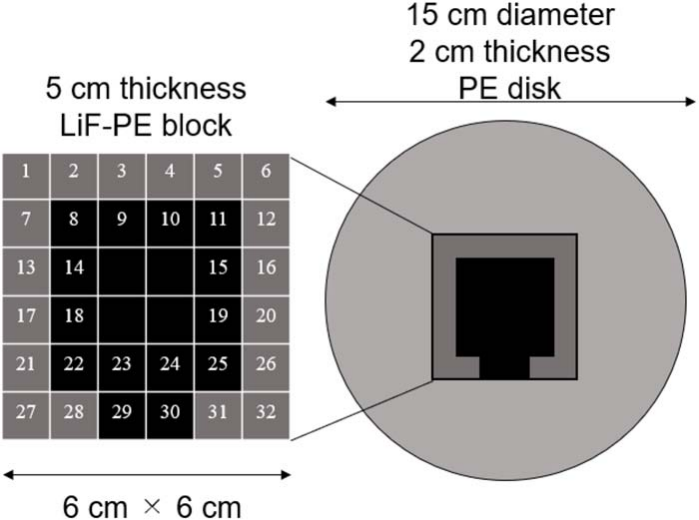
Shape of intensity modulator in IF-B.

The shape of the intensity modulator in IF-B for which the minimum tumor dose is the highest is shown in Fig. 4.

**Fig. 5:**
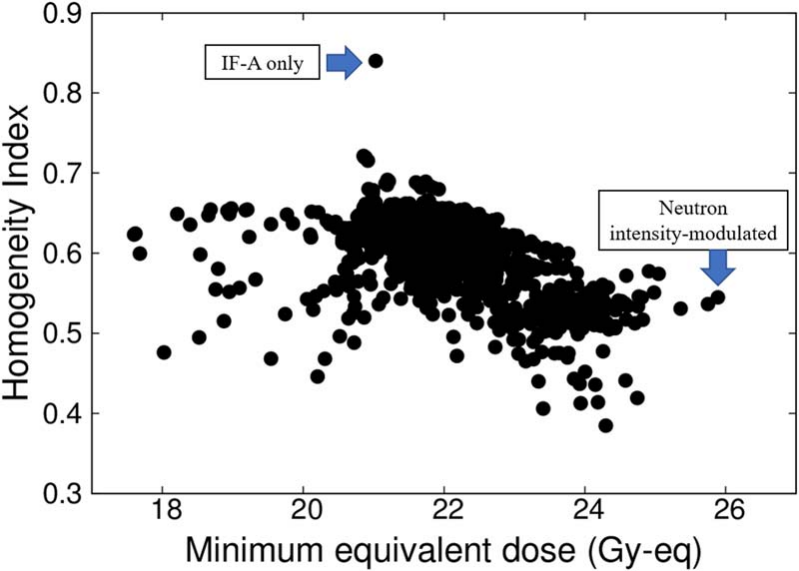
Plot of minimum tumor dose as a function of the HI.

A 5-cm thick LiF-PE block is inserted at the positions indicated in black (8, 9, 10, 11, 14, 15, 18, 19, 22, 23, 24, 25, 29 and 30) in Fig. 4. A 2-cm thick PE is placed at the positions shown in gray (1, 2, 3, 4, 5, 6, 7, 12, 13, 16, 17, 20, 21, 26, 27, 28, 31 and 32). Because the intensity modulator shape was determined by factors, such as the distance from the collimator surface to the tumor owing to the asymmetry of the head shape, the shape of the LiF-PE block was asymmetric along the AP direction. The irradiation time ratio between the irradiation fields A and B was 1:5. A plot of the minimum tumor dose as a function of HI is shown in Fig. 5. The plots for irradiation at a 1:5 irradiation time ratio using an optimally shaped intensity modulator and those for irradiation in irradiation field A are highlighted in the figure. This indicates that the combination of the determined IF-A and IF-B and irradiation time ratio yield the highest minimum tumor dose.

For all patterns, approximately, half a day was needed to run the calculations and to determine the intensity modulator shape and irradiation time ratio with the use of a single-core computer. A single Monte Carlo simulation requires ~5 min. If a multicore computer is used, the computation time can be reduced by the number of cores. SERA uses unique nuclear data and has a large voxel size of 1 cm^3^, which makes the computation time considerably short. Thus, there is some concern about the accuracy of the calculations. Therefore, the accuracy is ensured to some extent by comparing the thermal neutron flux at the surface in the evaluation area with the actual measurements. In fact, the thermal neutron flux at the evaluation point was confirmed by an irradiation test, and the SERA calculated value and actual measurement agreed with each other within an error of ~5%.

In the method proposed in reference [10], the time required to determine the shape depends on the skill of the operator. However, the method proposed in this study enables the intensity modulator shape and irradiation time ratio to be determined regardless of the skill of the operator once the tumor shape and irradiation direction have been determined.

### Evaluation of tumor dose distribution

The irradiation time, minimum tumor dose, HI for IF-A-only and intensity-modulated irradiation are listed in Table 1. The listed results show that the intensity-modulated irradiation improves the minimum tumor dose and the uniformity of the dose distribution, although the irradiation time is longer.

**Table 1:** Irradiation time, minimum tumor dose and HI for IF-A-only and intensity-modulated irradiation in a superficial tumor case with a diameter of 100 mm and a thickness of 10 mm

	Irradiation time (min)	Minimum tumor dose (Gy-eq)	HI
IF-A	67	21.0	0.84
Intensity-modulated	93	25.9	0.54

The dose and its distribution to brain using each irradiation method are as follows: on the one hand, for IF-A only, *D*_max_ was 11.4 Gy-eq, *D*_5_ was 6.1 Gy-eq and *D*_50_ was 0.9 Gy-eq. On the other hand, for IM, *D*_max_ was 11.5 Gy-eq, *D*_5_ was 6.5 Gy-eq and *D*_50_ was 1.3 Gy-eq. *D*_max_ denotes the maximum dose, and *D*_5_ and *D*_50_ denote the dose (Gy-eq) at which 5 and 50% of the skin or brain volumes are irradiated, respectively. The increased uniformity of thermal neutrons results in a slight increase in *D*_5_ and *D*_50_ relative to the brain.

**Fig. 6:**
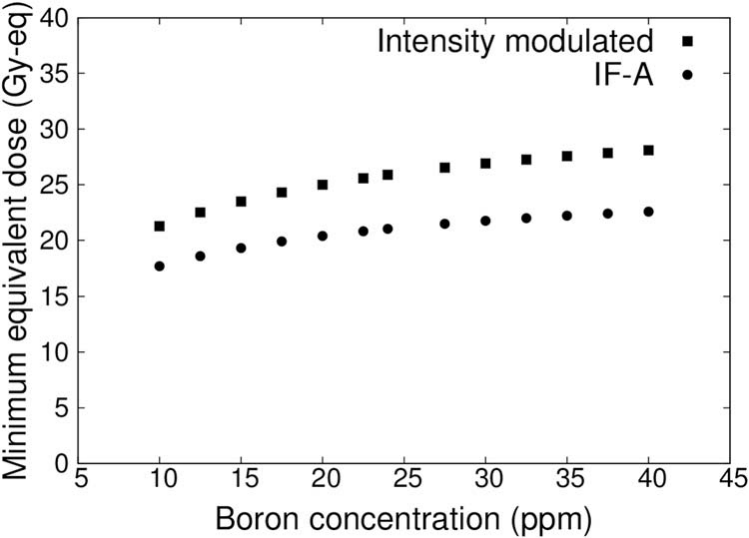
Minimum tumor dose at varying blood–boron concentrations for a superficial tumor with a diameter of 100 mm and thickness of 10 mm.

Figure 6 shows the plot of the minimum tumor dose as a function of blood–boron concentrations for IF-A-only and intensity-modulated irradiation. In both cases, the minimum tumor dose decreased as the blood–boron concentration decreased. However, the minimum tumor dose may be less than 20 Gy-eq when treated with IF-A. On the other hand, more than 20 Gy-eq is required for tumor control in a single irradiation [17, 18]. Therefore, treatment with IF-A alone may not provide sufficient therapeutic effect. By contrast, in the case of intensity-modulated irradiation, the minimum tumor dose was >20 Gy-eq even when the blood–boron concentration was approximately equal to 10 ppm. Thus, it is expected that treatment can be reliably performed even if the blood–boron concentration is below the planned level at the start or during the treatment.

**Fig. 7:**
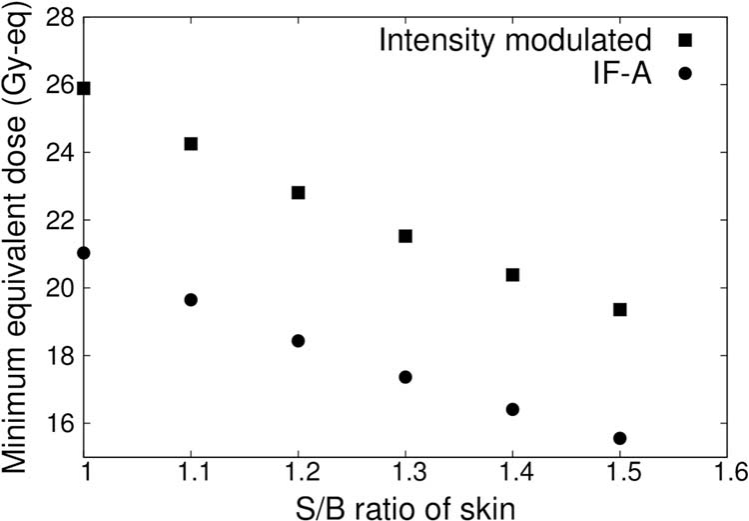
Minimum tumor dose at different S/B ratios for a superficial tumor with a diameter of 100 mm and thickness of 10 mm.

Figure 7 shows the minimum tumor dose when the S/B ratio of normal skin is varied from 1.0 to 1.5 for IF-A only and intensity-modulated irradiation. When the S/B ratio of normal skin was increased, the irradiation time was reduced and the dose to the tumor was reduced owing to the irradiation condition. The dose of normal skin was 12 Gy-eq. This showed that the minimum tumor dose was reduced in both cases (IF-A and intensity-modulated irradiation). In the case of IF-A, the minimum tumor dose was <20 Gy-eq if the S/B ratio of normal skin was >1.1. However, when intensity-modulated irradiation was used, the minimum tumor dose was 20 Gy-eq even when the S/B ratio was 1.5.

We have demonstrated the effectiveness of intensity-modulated irradiation with the use of the proposed method for superficial tumors with diameters of 100 mm and thicknesses of 10 mm, which are relatively shallow and wide, by determining the intensity modulation and irradiation time ratio.

### Irradiation test

**Fig. 8:**
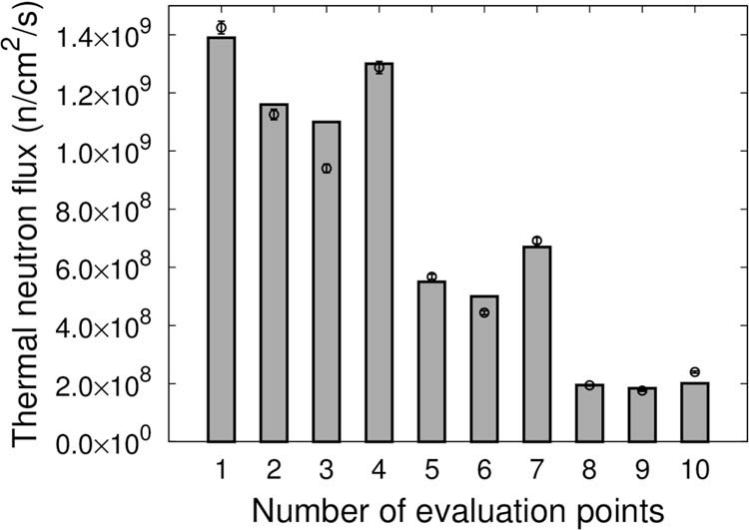
Thermal neutron flux at each evaluation point of IF-A.

Figure 8 shows the thermal neutron flux at each evaluation point for IF-A. The measured values are represented as points, and the values calculated using SERA are represented as bars. The thermal neutron flux distribution for IF-A was the highest at the irradiation center and was the lowest at the edges. The intensity of the thermal neutron flux at the evaluation points 5–7, ~5 cm away from the irradiation center (Evaluation Point 1), was approximately half. The uniformity of the thermal neutron flux for IF-A was *u_A_* = 33.2.

**Fig. 9:**
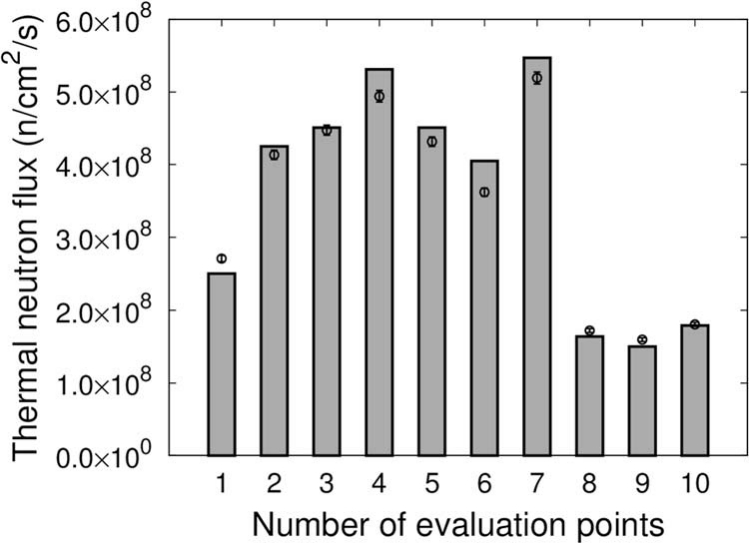
Thermal neutron flux at each evaluation point of IF-B.

Figure 9 shows the thermal neutron flux at each evaluation point for IF-B. The measured values are represented as points, and the values calculated using SERA are represented as bars. The distribution of the thermal neutron flux for IF-B was higher at the edges, ~5 cm away from the center of irradiation, compared with the center of irradiation. The uniformity of the thermal neutron flux for IF-A was *u_B_* = 14.5. By overlapping IF-A, which has a high-thermal-neutron flux distribution at the center of the irradiation field and a low thermal neutron flux distribution at the edges, with IF-B, which has a high thermal neutron flux distribution at the edges compared with the center at an appropriate time ratio to produce an intensity-modulated irradiation field, a uniform thermal neutron flux distribution can be generated.

The thermal neutron flux ${F}_{\mathrm{Intensity}\ \mathrm{Modulate}}$ for the intensity-modulated irradiation (IF-A and IF-B at a time ratio of 1:5) is defined based on the following equation:


$$ {F}_{\mathrm{Intensity}\ \mathrm{modulate}}=\frac{F_{\mathrm{A}}+5\times{F}_{\mathrm{B}}}{6}, $$

where ${F}_{\mathrm{A}}$ is the thermal neutron flux for IF-A and ${F}_{\mathrm{B}}$ is the thermal neutron flux for IF-B.

**Fig. 10:**
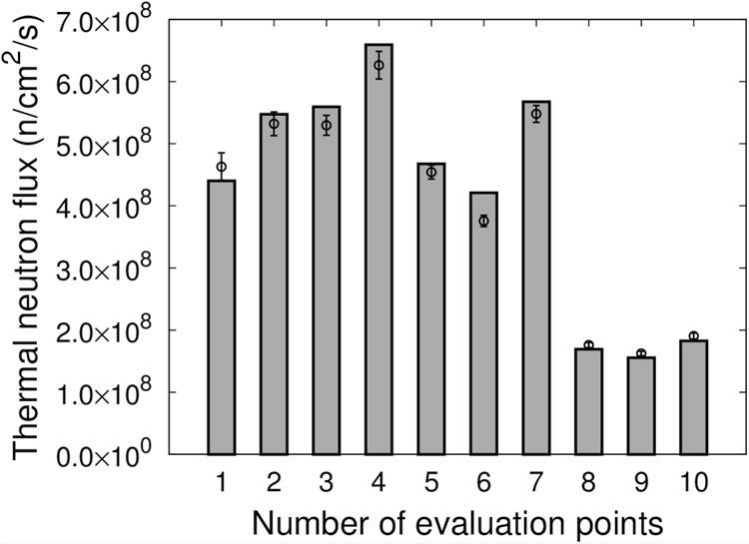
Thermal neutron flux of the intensity-modulated irradiation at each evaluation point.

Figure 10 shows the thermal neutron flux ${F}_{\mathrm{Intensity}\ \mathrm{modulate}}$ of the intensity-modulated irradiation at each evaluation point. The measured values are represented as points, and the values calculated using SERA are represented as bars. The results indicate that intensity-modulated irradiation is effective in creating a relatively uniform thermal neutron flux distribution over an area of ~10 cm in diameter at Evaluation Points 1–7. The uniformity of the thermal neutron flux was *u*_IM_ = 12.4, which corresponds to a 62% improvement in uniformity compared with IF-A only. The results for the SERA calculations were determined to be in good agreement with the actual measurements.

Hu *et al*. [19] demonstrated that, when epithermal neutrons are injected into the phantom, SERA is insufficient for evaluating the skin regions wherein thermal neutrons change rapidly owing to the large voxel size. Conversely, in the present study, the thermal neutrons were moderated to some extent in IF-A and IF-B; therefore, the thermal neutron flux in the voxel was less variable and the uncertainty in the skin dose due to the large voxel size was less than that in the case of direct injection of epithermal neutrons. Although it was difficult to verify the influence of voxel size on the uncertainty in the skin dose in this study, we confirmed the validity of SERA for skin doses by comparing the distributions of thermal neutron flux, which significantly affects skin doses, with actual measurements. Direct dose measurements are difficult. Thermal neutron fluxes at the skin surface were measured experimentally and compared with SERA calculations. The main dose component of BNCT is the boron dose. The boron dose is also dependent on the thermal neutron flux. Therefore, as the measured and calculated thermal neutron fluxes agreed, we consider this to be a dose assurance to some extent. Based on these findings, it was established based on experimental measurements on a tumor model in the irradiation field that a uniform thermal neutron flux distribution could be formed over an area with an approximate diameter of 10 cm. Thus, the method developed in this study to automatically determine the combination of intensity modulators and irradiation time ratio was validated.

### Adaptation to other cases

**Fig. 11:**
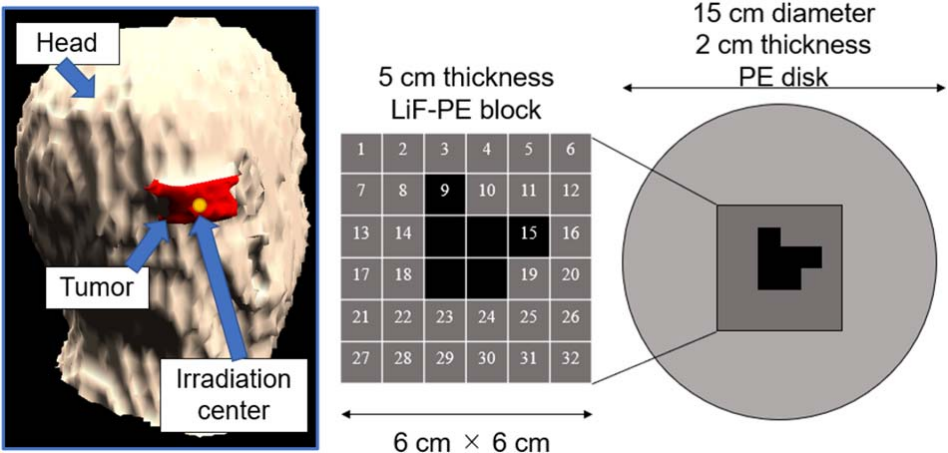
Tumor model and shape of the intensity modulator.

We evaluated the effectiveness of the proposed method in two cases. Figure 11 shows the first case of the tumor model and shape of the intensity modulator. The irradiation center corresponds to the center of the GTV.

A 5-cm thick LiF-PE block was inserted at the positions indicated in black (9 and 15) in Fig. 4. A 2-cm thick PE was placed at the positions shown in gray (except 9 and 15). The shielded area was small because the tumor model was small. The LiF-PE block was unbalanced because the intensity modulator shape reflects the nonuniformity of the tumor depth and head shape as observed from the beam.

The irradiation time, minimum tumor dose and HI for IF-A-only and the intensity-modulated irradiation are listed in Table 2. Listings show that the irradiation time increases by ~10 min, but the minimum tumor dose and HI are improved.

**Table 2:** Irradiation time, minimum tumor dose and HI for IF-A-only and the intensity-modulated irradiation in a relatively small tumor case

	Irradiation time (min)	Minimum tumor dose (Gy-eq)	HI
IF-A	83	33.1	0.65
Intensity-modulated	94	38.4	0.52

**Fig. 12:**
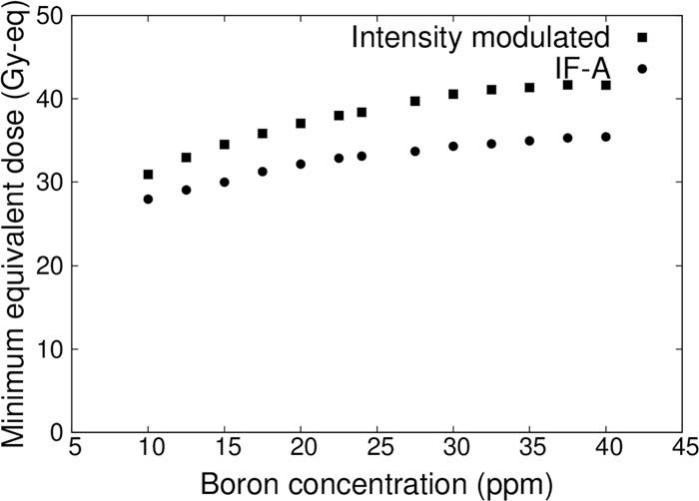
Minimum tumor dose at varying blood–boron concentrations for a relatively small tumor.

Figure 12 shows the minimum tumor dose for varying blood–boron concentrations for IF-A-only and intensity-modulated irradiation. This shows that the minimum tumor dose decreased when the blood–boron concentration decreased in both IF-A and intensity-modulated irradiation cases.

**Fig. 13:**
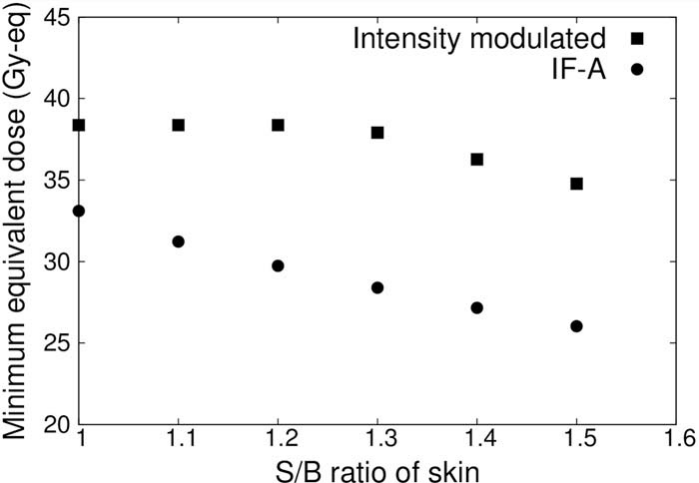
Minimum tumor dose at varying S/B ratio for a relatively small tumor.

Figure 13 shows the minimum tumor dose when the S/B ratio of normal skin was varied from 1.0 to 1.5 in the IF-A only and intensity-modulated irradiation cases. In these cases, the minimum tumor dose was reduced as the S/B ratio of the skin increased. In the IF-A case, the S/B ratio increased because the skin dose was the limiting factor, and the tumor dose decreased monotonically. By contrast, in the case of intensity-modulated irradiation, the minimum tumor dose did not decrease until the S/B ratio was equal to 1.2. This is because the skin dose was <12 Gy-eq and the irradiation time was determined by the brain dose limit of 15 Gy-eq in the irradiation conditions.

**Fig. 14:**
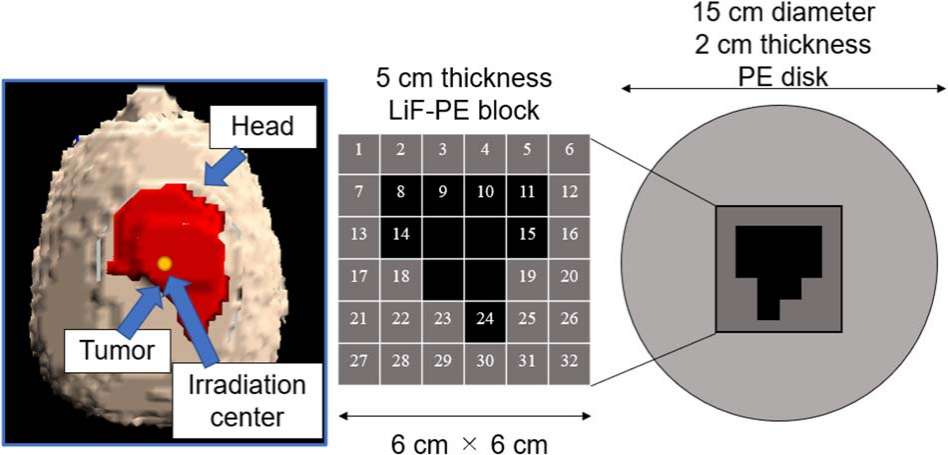
Tumor model and shape of the intensity modulator in an asymmetrical tumor case.

Figure 14 shows the second case of the tumor model and shape of the intensity modulator.

A 5-cm thick LiF-PE block was inserted at the positions indicated in black (8, 9, 10, 11, 14, 15 and 24) in Fig. 4. A 2-cm thick PE was placed at the positions shown in gray (except 8, 9, 10, 11, 14, 15 and 24).

The irradiation time, minimum tumor dose and HI for IF-A-only and intensity-modulated irradiation are shown in Table 3. The listings in Table 3 indicate that the irradiation time increases by ~25 min, but the minimum tumor dose and HI are improved.

**Table 3:** Irradiation time, minimum tumor dose and HI for IF-A-only and intensity-modulated irradiation in an asymmetrical tumor case

	Irradiation time (min)	Minimum tumor dose (Gy-eq)	HI
IF-A	65	20.7	0.89
Intensity-modulated	90	26.3	0.58

The developed method used for the determination of the intensity modulator shape and irradiation time ratio and for performing intensity-modulated irradiation was effective even for asymmetric tumors.

These results indicate that the proposed method is effective for any tumor shape and that intensity-modulated irradiation improves the minimum tumor dose and uniformity of the dose distribution.

## DISCUSSION

We developed a method to automatically determine the irradiation time ratio and the shape of the intensity modulator. This facilitated the generation of uniform intensity-modulated irradiation with a therapeutic dose distribution for tumors of various shapes. In the case of a relatively shallow and widely spreading tumor with a diameter of 100 mm and a thickness of 10 mm, it was possible to deliver an appropriate treatment dose even when the blood–boron concentration was low and the skin S/B ratios were high. For tumors with relatively small sizes, such as 40 × 40 mm, with thicknesses equal to 20 mm, intensity-modulated irradiation could deliver increased doses to the tumor, while it can minimize the dose to normal tissue, compared with single irradiation. Therefore, independent of the tumor size, intensity-modulated irradiation can potentially facilitate superior treatment outcomes compared with existing irradiation methods.

In the proposed method, the intensity modulator used for IF-A was a PE disk that enhanced the thermal neutron flux on the skin’s surface to treat superficial tumors. The optimal shape of the intensity modulator for IF-B was then determined with the use of a Monte Carlo simulation. However, it has been reported that the dose distribution to tumors deep inside the body can be improved by designing an intensity modulator inside the collimator [20]. Therefore, it may be possible to develop an effective intensity-modulated irradiation method for the treatment of deep-seated tumors by varying and combining the intensity modulators used for IF-A and IF-B.

It is also known that BNCT has a longer irradiation time than X-ray therapy. This raises concerns about the effects on dose distribution that are associated with errors in body positioning and patient body movements during irradiation [21, 22]. The intensity-modulated irradiation method improves the dose distribution by delivering a uniform thermal neutron flux to the tumor. The thermal neutron flux intensity at the edges of the tumor is comparable to that at the center. Therefore, it is expected to reduce the effects of errors owing to position setting and patient body movements.

Although intensity-modulated irradiation improves the dose distribution, the irradiation time is long. To apply the intensity-modulated irradiation method, irradiation must be completed within 1 h owing to the protocol of the boron drug administration. The time required to change the intensity modulator and patient’s positional settings must also be considered. Therefore, increasing the neutron intensity of the accelerator-based neutron source is necessary to implement the intensity-modulated irradiation method.

In terms of dose distribution, flattening the distribution of thermal neutron fluence will also homogenize the boron dose in the normal tissue distribution. Although the proportion of boron dose is not as large as that of the tumor dose, the normal tissue dose is also more homogenized than with conventional one-port irradiation, which may result in more areas with dose values close to the maximum dose value. In this study, *D*_5_ and *D*_50_ were also evaluated for brain doses and increased. When the developed IM technique will be used in the future, the distribution of normal tissue should be carefully evaluated, and the treatment protocol may also need to be improved.

## CONCLUSION

We created 424 patterns of source data in IF-B and performed calculations for all these patterns. Furthermore, for each result, the minimum tumor dose and HI were calculated when IF-A overlapped with the irradiation time ratios of 1:2, 1:3, 1:4 and 1:5. We automated the above process. The automated process determined the combination of IF-A and IF-B using the highest minimum tumor dose distribution and irradiation time ratio. Therefore, we developed a SERA-based method to automatically determine the optimal combination of intensity modulation and the irradiation time ratio to facilitate the treatment of superficial tumors in BNCT. The minimum tumor dose and the uniformity of the tumor dose distribution were improved by applying this method to a relatively shallow and widely spreading tumor model. Furthermore, the validity of the method was established based on an irradiation test by confirming that the thermal neutron flux was uniform in the defined tumor region. In addition, the method was applied to relatively small tumors and asymmetric tumors. The proposed method is expected to enable the uniform irradiation of tumors of any shape. 

## CONFLICT OF INTEREST

The authors declare that there are no conflicts of interest.

## FUNDING

This work was supported by the JST SPRING (grant number JPMJSP2110).

## DATA AVAILABILITY

The datasets generated and/or analyzed during the current study are not publicly available due to including source data for accelerator neutron sources. Still, they are available from the corresponding author on reasonable request.
